# Underweight Is an Independent Risk Factor for Renal Function Deterioration in Patients with IgA Nephropathy

**DOI:** 10.1371/journal.pone.0162044

**Published:** 2016-09-09

**Authors:** Yan Ouyang, Jingyuan Xie, Meng Yang, Xiaoyan Zhang, Hong Ren, Weiming Wang, Nan Chen

**Affiliations:** Department of Nephrology, Institute of Nephrology, Ruijin Hospital, Shanghai Jiao Tong University School of Medicine, Shanghai, 200025, China; Peking University First Hospital, CHINA

## Abstract

Studies on the relationship between body mass index (BMI) and renal progression in IgA Nephropathy (IgAN) were limited, especially for underweight patients with IgAN. To elucidate the clinical features and effect of underweight on renal function deterioration in this disease, we recruited IgAN patients with diagnostic age ≥18 years old and a baseline estimated glomerular filtration rate (eGFR) ≥15 ml/min/1.73m^2^ from our center between 1985 and 2014. Patients secondary to systemic diseases or follow-up less than 6 months were excluded. All patients’ clinical data at renal biopsy and during follow-up were recorded. Renal outcome was defined as end-stage kidney disease (ESRD). Baseline body mass index (BMI) was calculated by weight (kg) over squared height (m^2^). According to WHO Asian guideline, BMI was categorized as follows: <18.5kg/m^2^ (underweight), 18.5–22.99kg/m^2^ (normal weight), 23–27.49kg/m^2^ (overweight) and obese (≥27.5 kg/m^2^). Of 930 primary IgAN patients enrolled in this study, mean age at renal biopsy was 37.6 years and 49.2% were men. Totally, 114 (12.3%) ESRD occurred after a mean follow-up of 47.1 months. More ESRD happened in underweight patients (17.3%) compared to patients with normal weight (13.2%), overweight (11.0%) or obesity (9.5%). By multivariate Cox regression analysis, underweight was independently associated with a higher risk of ESRD after adjustment for demographic characteristics and clinical variables (HR: 3.5, 95% CI: 1.3–9.5, *P* = 0.01) comparing to normal weight. Underweight patients had lower hemoglobin, serum uric acid, triglycerides, cholesterol and lymphocyte counts than patients with normal weight. Furthermore, BMI was positively correlated with serum C3 (r = 0.25, *p* <0.001). Our research finds that underweight is an independent risk factor for kidney disease progression in IgAN, which might be associated with malnutrition status and decreased C3 levels.

## Introduction

Epidemiological studies have shown that IgA nephropathy (IgAN) is the most frequent type of primary glomerulonephritis worldwide, and represents the leading cause of end-stage renal disease (ESRD) in Asian populations [1–4]. Although the prognosis is highly variable, approximately 30–50% of patients develop ESRD within 20 years after diagnostic renal biopsy [5]. The identification of patients with IgAN, who are at a higher risk of ESRD, would be important for clinical practice. Therefore, there is a compelling need for determining more indicators to predict prognosis, in order to effectively facilitate planning for service provision and reduce risk in this population.

Mean body mass index (BMI) levels and the prevalence of obesity continue to increase worldwide [6, 7]. More attention has been given to the influence of obesity on chronic kidney disease (CKD). Several mechanisms involved in the association between obesity and CKD have been proposed, and weight loss has been recommended [8, 9]. Obesity is a significant predictor for ESRD in CKD patients [10–12]. Intriguingly, a large international prospective cohort study of 16,720 hemodialysis patients revealed that obesity was associated with reduced risk of death [13]. Furthermore, this paradox has not only observed in hemodialysis patients. In a CKD cohort study of 726 patients, Ricardo *et al*. [14] found that BMI <20 kg/m^2^ was associated with higher risk of all cause mortality. This finding was supported by various CKD studies, in which lower BMI was associated with greater mortality [15–18].

This differed from the CKD composition in Western countries, in which the main ingredient was diabetic nephropathy. In Asia, especially in China, IgAN is the predominant component of CKD. Only a few studies have focused on the relationship between BMI and renal outcome in IgAN. Among these studies, most of them revealed that excessive BMI was a risk factor for progressive renal disease in IgAN [19–23]. Some researchers presented that underweight contributed to cardiovascular events and death [14, 24]. However, few have elucidated the association of underweight and renal outcome, particularly in individuals with IgAN.

Therefore, we designed a large-scale study with long-term follow-up, in order to define whether underweight plays a role in renal disease progression in Chinese patients with primary IgAN. In addition, we also investigated the potential mechanisms involved.

## Materials and Methods

### Research design and study participants

**Fig 1:**
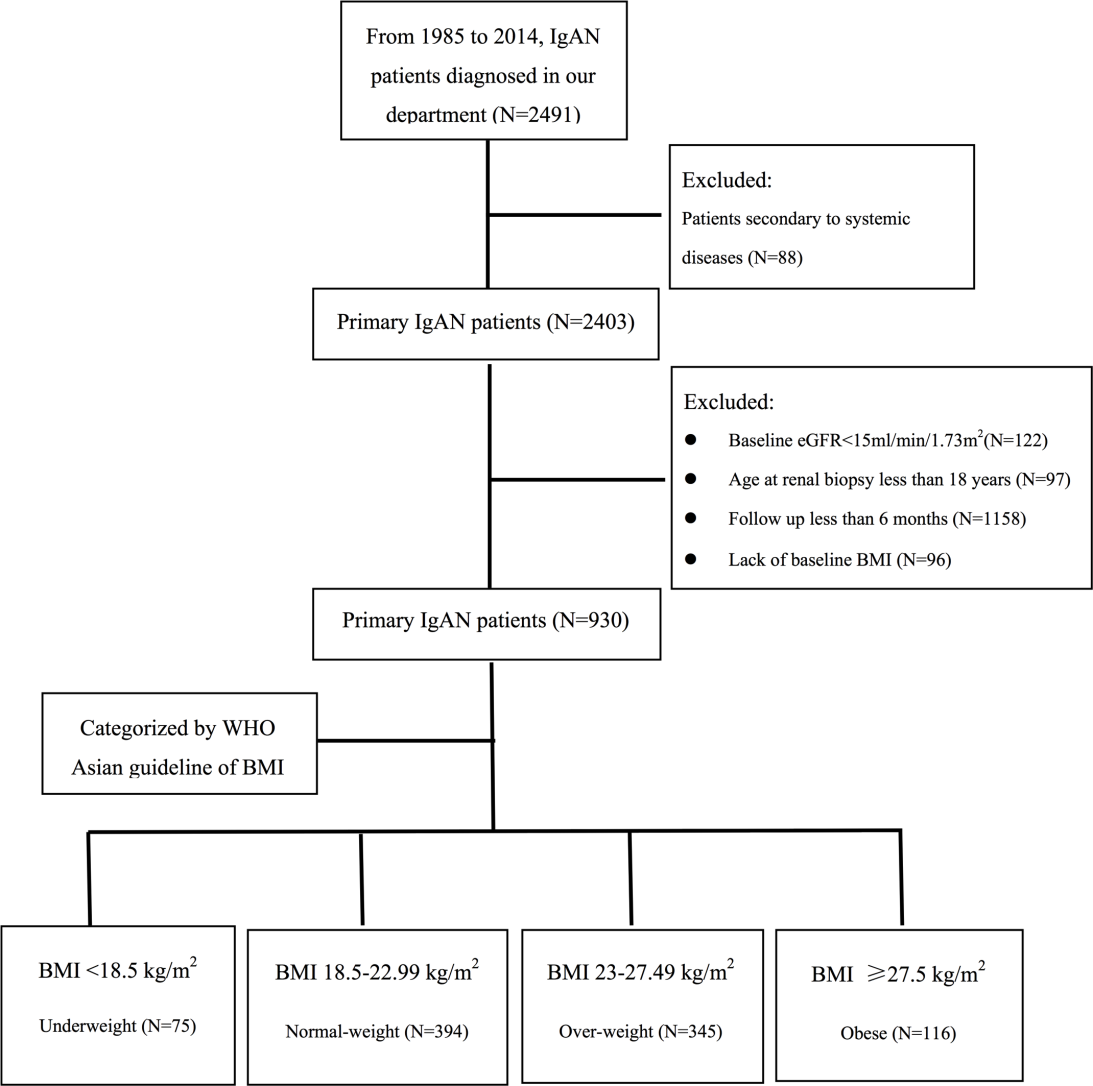
Screening, enrollment, and patients eligible for the analysis. The exclusion criteria were show as follows: secondary to system disease, baseline eGFR value of less than 15ml/min/1.73^2^, biopsy age less than 18 years old, follow-up less than 6 months and lack of baseline BMI were excluded.

In 2016, a cohort study was conducted to investigate the association between underweight and the development of IgAN. The records of 2,491 patients with biopsy-proven IgAN, who diagnosed in our center from 1985 to 2014, were retrospectively collected. Among these cases, eighty-eight patients secondary to systemic disease, one hundred and twenty-two patients with baseline eGFR value less than 15 m/min/1.73m^2^, ninety-seven patients with biopsy age less than 18 years old, eleven hundred and fifty-eight patients with a minimum follow-up time of six months and ninety-six patients with absence of baseline BMI were excluded (Fig 1). Therefore, a total of 930 patients were recruited in this study. Clinical data at renal biopsy were recorded at inpatient visits, and the follow-up data were collected at outpatient visits. This study was performed in accordance with the Declaration of Helsinki and approved by the Ethics Committee of Ruijin Hospital, Shanghai Jiaotong University. All subjects provided written informed consent.

### Study outcomes

The main predefined study outcome for the present analysis was the occurrence of ESRD. ESRD was defined as eGFR<15 ml/min/1.73m^2^ with the need for renal replacement therapy (dialysis or renal transplantation). Patients who were loss to follow up or reached the endpoints were not disclosed.

### Definition and classification of BMI

Baseline BMI was defined as weight in kilograms divided by the square of the height in meters. Due to the ethnic specificity of BMI, we chose the cut points in the WHO Asian standard [25]. BMI categories were as follows: underweight (<18.5kg/m^2^), normal weight (18.5–22.99 kg/m^2^), overweight (23–27.49kg/m^2^) and obese (≥27.5 kg/m^2^).

### Demographic and clinical variables

Demographic and clinical variables of all patients were collected as previous descriptions [26]. All variables were available to clinicians, and these were accurately measured in a standardized and reproducible way. Complement component 3 (C3) was measured by immunoturbidimetry using the IMMAGE Immunochemistry System and corollary reagent (Beckman Coulter, USA). The normal reference range of serum C3 was defined as 85–193 mg/dl, which was recommended by the supplier. Serum levels of high sensitive C-reactive protein (hsCRP) were measured by latex-enhanced immunoturbidimetry (Roche Diagnostics, Germany). Two types of candidate clinical indicators were considered: (1) demographic data such as age, gender, systolic blood pressure (SBP), diastolic blood pressure (DBP) and BMI; (2) laboratory parameters such as serum creatinine (Scr), estimated glomerular filtration rate (eGFR), urine acid (UA), albumin (Alb), hemoglobin (Hb), lymphocyte count, 24-hour urine protein, serum cholesterol, serum triglycerides, serum C3, white blood cell (WBC) and hsCRP. C3 hypocomplementemia (hypoC3) was defined as serum C3 level less than 85mg/dl. eGFR was determined from serum Scr levels by applying the Chronic Kidney Disease Epidemiology Collaboration (CKD-EPI) equation [27]. Stages of CKD were based on the Kidney Disease Outcomes Quality Initiative (K/DOQI) practice guidelines[28].

### Statistical analysis

The distributions of quantitative variables were assessed for normality. Continuous data were expressed as mean ± standard deviation (SD) or median (range). For normally distributed variables, two groups were compared by *t*-test. Mann-Whitney *U*-test was used to compare two groups of non-parametric variables. Categorical data were expressed as frequencies or percentages (%), and compared by using standard chi-squared test. Missing data were defined as default value. Probabilities of cumulative renal survival curves were generated by the Kaplan-Meier method, and compared by the log-rank test. Patients who reached the time of renal events or were loss to follow-up were not disclosed. Cox regression analysis results were expressed as hazard ratios (HRs) with 95% confidence intervals (CIs). Logistical regression analysis results were expressed as odd ratios (ORs) with 95% CIs. Correlation analysis determined whether there was a possible linear relationship between two variables. Statistical analysis was performed using SPSS version 22.0 (SPSS Inc, Chicago, Illinois, USA). *P*-values of <0.05 were considered statistically significant.

## Results

### Patients’ demographic and clinical characteristics

The baseline data of 930 eligible cases (458 males and 472 females, 1:1.03) were shown in Table 1. Average age at biopsy was 37.6 ± 11.8 years, and mean follow-up time after renal biopsy was 47.1 months (range: 6–246 months). During the follow-up period, 12.3% (114/930) subjects developed ESRD. Univariate Cox analysis revealed the effect of baseline clinical indicators on progression to ESRD (Table 1).

**Table 1:** Baseline clinical variables of IgAN patients and analysis of those indicators affect ESRD by univariate Cox regression.

Baseline variables	Total	Hazard Ratio (95% CI)	P value
	(N = 930)		
Age at biopsy, years	37.57±11.83	1.01(0.99–1.02)	0.55
Female gender, n (%)	472/930 (50.75%)	0.55(0.37–0.80)	0.002
Body mass index, kg/m^2^	23.24±3.63	0.96(0.91–1.02)	0.18
Urine protein, g/24 h	1.17(0.02–10.83)	1.27(1.17–1.37)	<0.001
Systolic blood pressure, mmHg	128.22±17.35	1.02(1.01–1.03)	<0.001
Diastolic blood pressure, mmHg	80.92±12.10	1.03(1.02–1.04)	<0.001
eGFR, ml/min/1.73m^2^	72.84±32.81	0.94(0.93–0.96)	<0.001
Serum albumin, g/L	34.76±6.67	0.95(0.92–0.97)	<0.001
Hemoglobin, g/L	128.16±20.22	0.98(0.97–0.98)	<0.001
Urine Acid, μmol/l	385.15±104.91	1.007(1.005–1.008)	<0.001
Lymphocyte count, ×10^9^/L	2.31±0.78	0.63(0.47–0.86)	0.003
Serum cholesterol, mmol/L	5.36±1.70	1.07(0.96–1.19)	0.24
Serum triglycerides, mmol/L	1.89(0.45–11.68)	1.08(0.96–1.22)	0.2
Serum C3, mg/dL	104.26±26.54	0.99(0.98–0.999)	0.03
Renal deposition of C3, n (%)	499/645 (77.36%)	0.84(0.46–1.52)	0.57
C3 hypocomplementemia, n (%)	181/728 (24.86%)	1.33(0.84–2.10)	0.22
White blood cell, ×10^9^/L	7.55±2.22	0.95(0.86–1.05)	0.33
High sensitive C reactive protein, mg/L	0.65(0.02–123.82)	1.01(0.98–1.03)	0.58

Abbreviations: C3: complement component 3; eGFR: estimated glomerular filtration rate. According to their normality, continuous data were expressed as mean ± standard deviation (SD) or median (range). Categorical data were expressed as percentages (%). univariate Cox regression was used to evaluate the association of baseline variables with the deterioration to ESRD.

### Distribution of BMI

**Fig 2:**
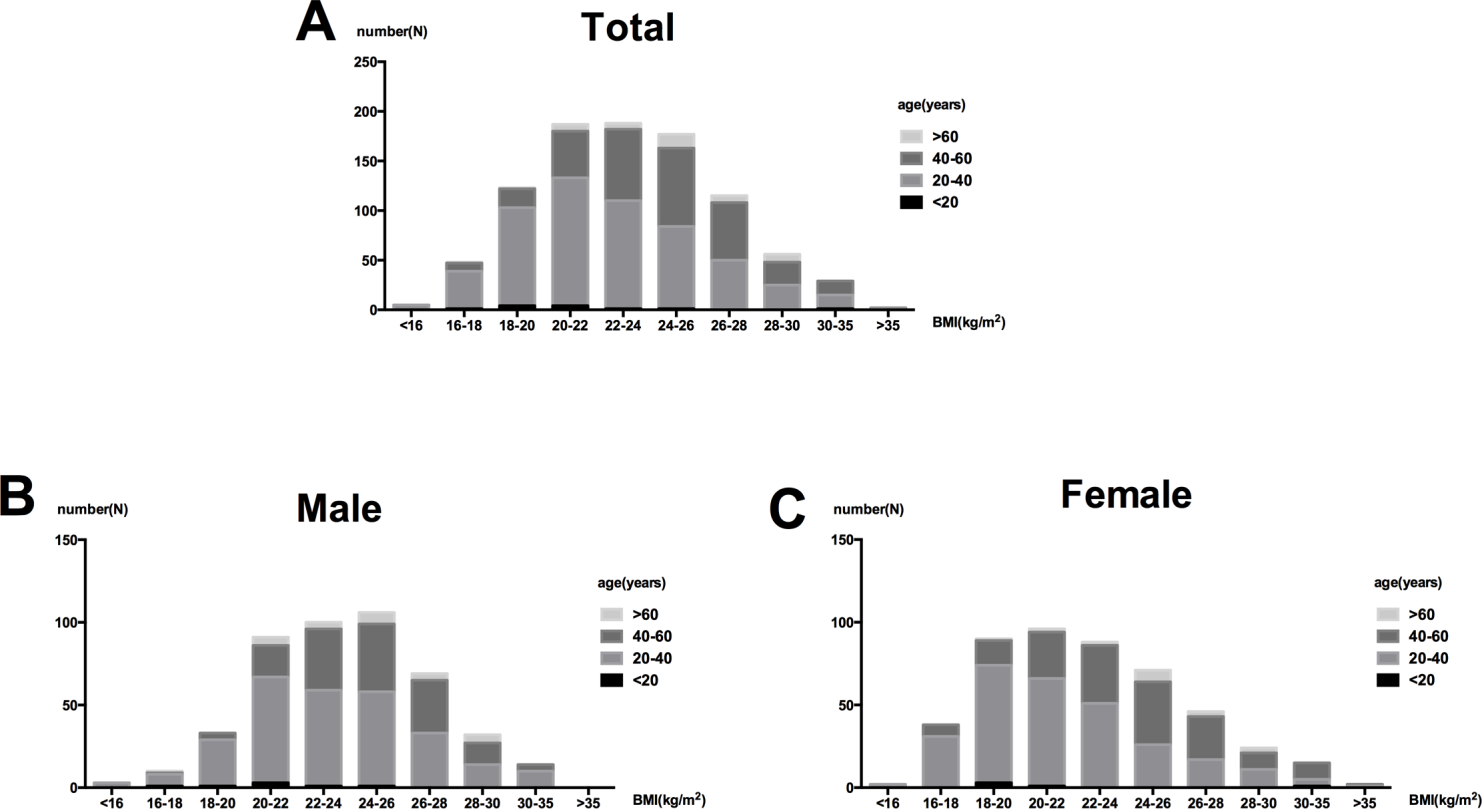
Distribution of BMI in 930 IgAN patients. (A) BMI goups were separated by age categories in all patients with IgAN, and then divided into males (B) and females(C).

For all patients, the distribution peak of BMI shifted to the left (Fig 2A). Patients with BMI > 27.5kg/m^2^ only accounted for 12.5% of the total subjects (14.0% in males and 11.0% in females). Meanwhile, the incidences of underweight cases in females were more than that in males (12.7% vs. 3.3%). Bars of the BMI were divided by age categories (Fig 2). Patients who had a small BMI tended to be young.

### BMI and renal outcome

In order to confirm the association of BMI with ESRD in IgAN patients, Cox regression was used to analyze this continuously variable. There was no significant difference in the univariate Cox analysis. However, this difference became significant by the additional adjustment for age and gender (HR: 0.94, 95% CI: 0.88–1.00, *P* = 0.049). Even in a full-adjusted model of demographic variables and clinical indicators, lower BMI continued to increase the risk of ESRD with a significant difference (HR: 0.92, 95% CI: 0.86–0.98, *P* = 0.011).

**Fig 3:**
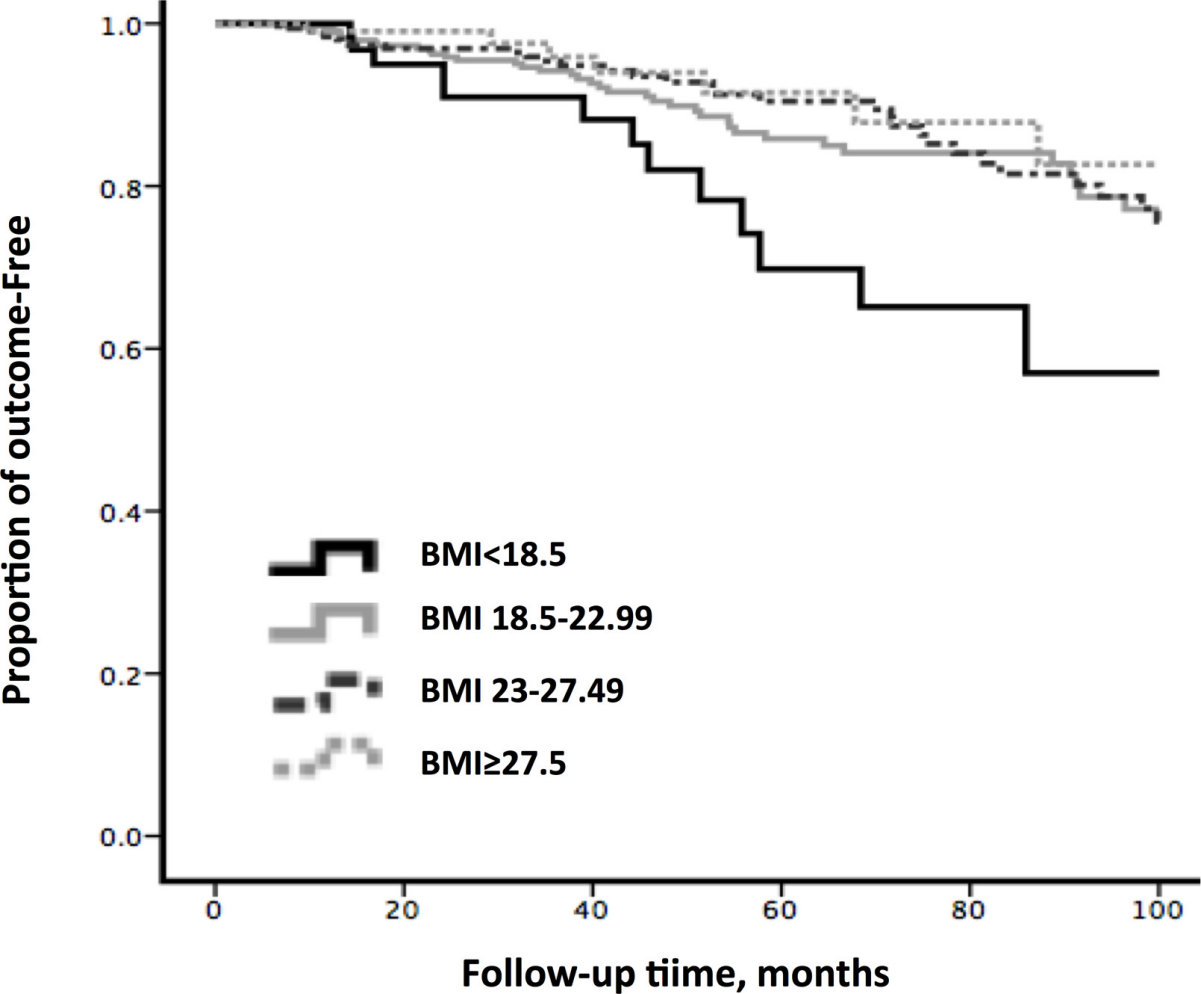
Kaplam-Meier Survival analysis of ESRD occurred in different BMI groups. The mean ESRD-free time was 79.51±4.70, 89.90±1.55, 91.34±1.52 and 92.83±2.58 months for Underweight, Normal, Overweight and Obesity group, separately (p value = 0.03). Vertical tick lines indicate censor points.

Next, the effect of underweight on ESRD was examined in our patients. According to the WHO Asian classification for BMI, these were categorized into four BMI groups. However, the underweight group that presented better baseline eGFR and blood pressure continued to have the worse renal outcome, compared with the normal weight group (Table 2). As shown in Fig 3, Kaplan-Meier survival analysis revealed that mean ESRD-free time of patients in the underweight group (79.5 ± 4.7months) was significant shorter than the normal weight (89.9 ± 1.6 months), overweight (91.3 ± 1.5 months) and obesity (92.8 ± 2.6 months) groups (*P* = 0.03) during the follow-up period of 100 months.

**Table 2:** Compare baseline data among different BMI groups categorized by WHO Asian standard.

Baseline variables	BMI (kg/m^2^)			
	<18.5	18.5–22.99	23–27.49	≥27.5
	(N = 75)	(N = 394)	(N = 345)	(N = 116)
Age at biopsy, years	31.31±10.28 *	34.78±10.91	40.48±11.70 ***	42.42±12.02 ***
Female gender, n (%)	60/75(80.0%)***	216/394(54.8%)	144/345(41.7%)	52/116(44.8%)
BMI, kg/m^2^	17.50±0.81 ***	20.94±1.26	25.03±1.21 ***	29.41±2.96 ***
Urine protein, g/24 h	1.13(0.09–9.21)	1.01(0.04–10.83)	1.29(0.02–10.10)**	1.94(0.14–7.46)***
SBP, mmHg	122.45±18.29	125.59±17.32	129.30±16.09 **	137.59±16.71 ***
DBP, mmHg	76.89±13.35	79.24±11.90	81.89±11.69 **	86.30±11.09 ***
eGFR, ml/min/1.73m^2^	76.22±37.62	76.61±34.45	68.63±30.25 **	70.35±29.59
Alb, g/L	34.47±6.03	34.49±6.97	35.44±6.13	33.81±7.44
Hb, g/L	115.94±19.01 ***	125.15±19.66	131.90±19.64 ***	135.01±19.49 ***
UA, μmol/l	369.18±133.01	373.91±110.73	394.47±3.32 **	405.19±91.87 **
TG, mmol/L	1.28(0.46–4.91)	1.53(0.45–7.79)	2.24(0.48–7.55)**	2.60(0.65–11.68)***
Chol, mmol/L	5.00±1.43	5.29±1.71	5.34±1.67	5.92±1.79 **
Lymphocyte count, ×10^9^/L	2.25±0.81	2.31±0.80	2.29±0.71	2.42±0.87
Serum C3, mg/dL	92.35±25.53	98.12±24.11	109.56±25.92***	115.91±28.81***
Renal deposition of C3, n (%)	46/56(82.1%)	216/268(80.6%)	178/237(75.1%)	59/84(70.2%)
HypoC3, n (%)	29/60(48.3%)*	97/297(32.7%)	44/281(15.7%)***	11/90(12.2%)***
WBC, ×10^9^/L	7.25±2.27	7.43±2.37	7.58±2.03	8.07±2.10
hsCRP, mg/L	0.32(0.02–11.90)**	0.51(0.1–42)	0.89(0.05–123.82)**	1.16(0.20–75.00)***

Abbreviations: BMI: Body mass index; SBP: systolic blood pressure; DBP: diastolic blood pressure; Alb: albumin; Hb: hemoglobin; UA: uric acid; Chol: cholesterol; TG: triglycerides. HypoC3: C3 hypocomplementemia; WBC: white blood cell; hsCRP: high sensitive C reactive protein. Other three BMI groups compared with normal weight group, which was used as reference.

*, P value <0.05

**, P value <0.01

***, P value <0.001.

After a mean follow-up time of 47.1 months (range: 6–246 months), a total of 114 individuals (12.3%) reached the study endpoint. Incident ESRD cases in underweight patients (17.3%) were still more than that in normal weight (13.2%), overweight (11.0%) and obesity patients (9.5%). In univariate Cox analysis, compared with normal weight group, no significant association with ESRD was observed for overweight (HR: 0.9, 95% CI: 0.6–1.3, *P* = 0.5) or obese (HR: 0.8, 95% CI: 0.4–1.6, *P* = 0.6), while underweight had a powerful effect on the worse renal prognosis (HR: 1.9, 95% CI: 1.0–3.5, *P* = 0.04). Even when analyzed by multivariate Cox regression analysis, compare to the normal BMI group, the underweight group (HR: 2.1, 95% CI: 1.1–3.9, *P* = 0.02) continued to significantly increase the risk of ESRD in Model1. Similar results were also found in Model2 and Model3, which were consistent with the results above (Table 3).

**Table 3:** Hazard ratios and 95% CI presented different progressive risk in four BMI groups.

BMI	Model1		Model2		Model3	
(N = 930)	HR (95% CI)	P value	HR (95% CI)	P value	HR (95% CI)	P value
<18.5(N = 75)	2.08(1.12–3.86)	0.02	2.08(1.08–3.99)	0.03	3.51(1.29–9.52)	0.01
18.5–22.99(N = 394)	-ref-	-ref-	-ref-	-ref-	-ref-	-ref-
23–27.49(N = 345)	0.74(0.48–1.16)	0.19	0.54(0.34–0.86)	0.01	0.54(0.26–1.11)	0.09
≥27.5 (N = 116)	0.80(0.41–1.56)	0.51	0.69(0.35–1.35)	0.28	0.81(0.25–2.59)	0.72

Model 1 is adjusted by bioage and female gender, Model 2 is adjusted by 24h urine protein, eGFR in addition to variables in Model 1, Model 3 is adjusted by SBP, DBP, Alb, Hb, UA, TG, Chol, Lymphocyte count, serum C3, renal deposition of C3 and hypoC3 in addition to variables in Model 2.

Furthermore, compared to the normal weight group, significantly lower levels of hemoglobin (P<0.001), urine acid, serum triglycerides and cholesterol were also observed in the underweight group. These data strengthened the notion that the underweight group had the worse nutrition status (Table 2). In order to investigate whether worse nutrition status was associated with a higher level of inflammation, we analyzed baseline inflammation markers including hsCRP and WBC in our patients. We found that hsCRP and WBC levels increased when BMI increased, and both of these inflammation markers were lower in underweight group than in the other three groups (Table 2).

### Levels of complement in the different BMI groups

Serum C3 levels were compared among different BMI groups. Our data showed that the underweight group presented the lowest level of serum C3 (92.4 ± 25.5 mg/dL), followed by the normal weight (98.1 ± 24.1 mg/dL), overweight (109.6 ± 25.9 mg/dL) and obese (115.9 ± 28.8 mg/dL) groups. As the BMI decreased, serum C3 levels exhibited a descending trend. Interestingly, although no statistical significance of mesangial C3 deposition was found between the normal weight and underweight groups, its incidence in the underweight group remained higher than the normal weight group (Table 2).

In order to confirm the association between hypoC3 and underweight, our data revealed that the incidence of hypoC3 in the underweight group was higher than in the other three groups. There was a statistically significant difference between the underweight and normal groups. Then, we evaluated the risk of hypoC3 among these BMI groups by univariate logistic regression, and used the obesity group as the reference. Results suggested that the underweight group had the highest risk (OR: 6.7, 95% CI: 3.0–15.1, *P* <0.001), followed by normal weight (OR: 3.6, 95% CI: 1.8–7.0, *P* <0.001) and overweight (OR: 1.3, 95% CI: 0.7–2.7, *P* = 0.426) groups.

### Correlation analysis between the baseline BMI and complement levels or serum lipids

**Fig 4:**
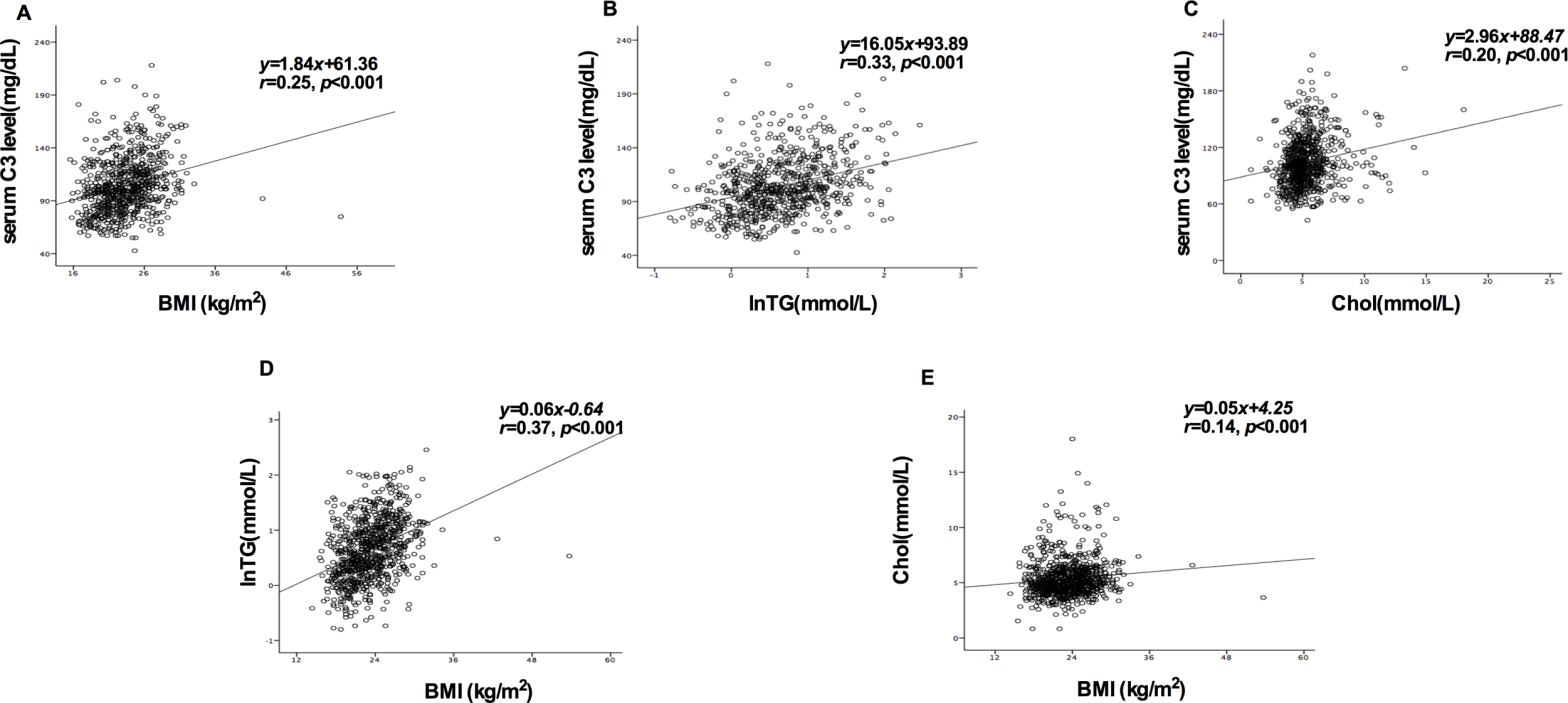
Correlation analysis assessed the relationship between any two indicators of BMI, serum C3, triglycerides and cholesterol. (A) Correlation between serum C3 levels and BMI at baseline; (B) Correlation between the normal conversion of triglycerides and serum C3 levels; (C) Correlation between cholesterol and serum C3 levels; (D) Correlation between BMI and normal conversion of triglycerides; (E) Correlation between BMI and cholesterol.

The correlation analysis indicated that there was a positive correlation between serum C3 and baseline BMI (r = 0.25, P<0.001). According to literature, triglycerides and cholesterol levels were associated with serum C3. In order to explore the mechanisms of the association between BMI and C3 in IgAN, the correlations between serum lipid and C3 or BMI were separately analyzed. Then, it was found that there was indeed a positive relationship between any of two factors among these (Fig 4).

### BMI and underlying disease

In order to confirm whether underweight is secondary to underlying diseases including hyperthyroidism and hepatitis virus B infection, we investigated the frequency of these conditions in the four BMI groups. Our data revealed that 0.3% (3/930) of cases had hyperthyroidism and 4.0% (37/930) of cases were carriers of hepatitis B virus in the underweight group. The differences in the rates of these two underlying diseases among the BMI groups were not significant (S1 Table).

## Discussion

To our knowledge, through this large cohort study of 930 primary IgAN patients with a mean follow-up period of 47.1 months, this study is the first to investigate the impact of underweight on the renal outcome in IgAN. Based on the reference value of the WHO Asian standard [25], our patients were divided into four groups: underweight, normal weight, overweight, and obesity groups. Intriguingly, we observed that the incidence of ESRD in the underweight group was higher than in other three BMI groups during the follow-up interval. In addition, the underweight group presented the shortest mean ESRD-free time among the four groups, as revealed by the survival curve analysis. Furthermore, Cox analysis data were also consistent with the above results, suggesting that underweight was an independent risk factor for progression to ESRD in IgAN.

Previously, excessive BMI has been reported as a risk factor for renal disease progression in IgAN patients in several studies [19–23]. A similar paradox was also found in former CKD studies on the association between BMI and adverse outcome. Some studies presented that obesity was associated with increased risk for the incidence of ESRD in CKD [10, 29]. Moreover, focal segmental glomerulosclerosis (FSGS) and glomerulomegaly were often observed among obese people [29–31]. In contrast, another reports have found that hemodialysis patients with low BMI had increased risk of mortality [13, 32]. In addition to patients undergoing dialysis, a study conducted by Kovesdy *et al*. [16] revealed that this inverse association also presented in CKD patients. In general, these studies have reported heterogeneous findings [33–35].

However, U-shaped associations of BMI with urinary albumin-to-creatinine ratio (uACR) had been reported. Data revealed that the level of uACRs in the lowest and highest BMI groups were higher than in the remaining BMI groups [36]. It was also widely accepted that the association between BMI and all-cause mortality was U-shaped with the lowest mortality between 22.5–25 kg/m^2^ [37]. Moreover, Muneyuki *et al*. [38] indicated that the U-shape association between BMI and proteinuria were independent of traditional cardiometabolic risk factors. Since low or high BMI is related to higher proteinuria risk and mortality, we reckoned that underweight might participate in renal disease progression in IgAN patients. Although an appropriate hypothesis for this paradox has not yet been proposed, our cohort study could not solve this problem either.

In fact, among the numerous complications of CKD, one of the most common conditions is the progressive loss of body protein mass and energy reserves [39]. Some cohort studies have mainly recruited younger patients, and indicated that BMI could better reflect the amount of muscle mass, when compared to waist–hip ratio, because ageing body composition generally changes and muscle mass shifts to fat mass accompanied by lower bone mass [40, 41]. Lower BMI reflects both low fat and low muscle mass. Our subjects were mostly young, in which only 4.7% (44/930) of the patients in this study were older than 60 years. This means that BMI was more suitable for our study.

As an important condition that appeared in approximately 20–50% of advanced CKD patients, protein–energy wasting could predict poor clinical outcome [39, 42]. Metabolic disorders in CKD patients led to exaggerate protein degradation rather than protein synthesis [40]. In particular, additional protein loss, as well as long-term moderate and severe proteinuria, would accelerate this deterioration. Since higher BMI was associated with reduced risk of cardiovascular death, obese subjects with proteinuric kidney disease may not be counseled for weight reduction [43]. In our study, most of our patients had proteinuria, in which 57.0% of these patients had proteinuria over 1 g/d. Additional protein loss due to proteinuria might be tolerated worse in patients with lower BMI, because lower protein reserve might lead to a more unfavorable outcome. This appears as a plausible explanation why underweight subjects with IgAN were more likely to become malnourished.

In the past, no single marker can be regarded as an ideal indicator to assess nutritional status [44]. In 2015, the European Society of Clinical Nutrition and Metabolism (ESPEN) Consensus Statement rendered a clear and simple format for the diagnosis of malnutrition. Due to strong global acceptance, a cut-off of 18.5 kg/m^2^ recommended by the WHO was unanimously decided and accepted as a criterion to diagnose malnutrition [45]. Our present results revealed Hb, UA, TG, Chol and lymphocyte count in the underweight group were lower than in the normal group. These data meant that underweight was a powerful risk factor for ERSD in IgAN patients, which was mainly due to malnutrition status. Malnutrition had already been reported that it would accelerate renal function progression [46]. Our results were in accordance to this.

From another perspective, based on the GWAS research on IgAN conducted by Kiryluk *et al*. [47], they suggested a multi-hit theory that indicated that the complement pathway activated by the renal deposition of antigen-antibody complexes (CIC) causes damage to kidney tissues. Several functional experiments [48–50] and genetic association studies [51, 52] have highlighted the pathogenic role of the complement pathway in IgAN. A lower serum C3 status was reported in patients with IgA nephropathy [53–55]. Moreover, Kim *et al*. [48] found that 19.2% of patients (66/343) have hypoC3. Furthermore, a Korean study [48] revealed that hypoC3 and mesangial C3 deposition were independent risk factors for the progression in IgAN. These findings confirm that low C3 does exist in IgAN patients and have potential clinical significance.

In univariate Cox regression, we found that lower C3 level was a risk factor for ESRD in IgAN. In addition, there was a positive correlation between BMI and C3 levels. This phenomenon suggests that underweight might be involved in the disease development by lowering serum C3 levels. It is well known that nutritional status is important for maintaining normal immune function. Worse nutrition status marked by a lower BMI will potentially lead to immune dysfunction including a decreased serum level of C3 [56]. Hence, we attempted to investigate the possible regulatory mechanisms between BMI and C3.

On one hand, the total complement and C3 may be at a critical level in patients with malnutrition. Furthermore, underweight patients might increase consumption of C3 by complement activation, owing to the microinflammatory state of undernutrition. On the other hand, we found that both C3 and BMI had a linear correlation with lipid levels, which was consistent with previous studies. Actually, triglyceride [57]and total cholesterol [58, 59] had already been reported that both of them were closely related to serum C3 levels. C3 is mainly secreted by the liver [60], but activated macrophages and adipose tissue also synthesizes C3 [61, 62]. As a result of the interaction of C3, factor B, and adipsin (factor D), adipocytes synthesize and secrete the fragment of C3 (C3a-des-Arg) that was also known as acylation-stimulating protein (ASP) [61]. ASP stimulates glucose uptake, activates diacylglycerol acyltransferase (DGAT), and inhibits hormone sensitive lipase (HSL), so as to promote triglyceride synthesis in adipocytes [63]. This complement mechanism plays an important role in regulating lipid metabolism and energy balance.

On the basis of this theory, we further analyzed and confirmed the relationship between BMI, serum C3 and lipid levels in our IgAN patients. The incidence of hypoC3 in the underweight group was significantly higher than in the normal weight, overweight and obesity groups. The possible mechanism might be that lower BMI reflects the lower blood lipid, which is induced by the decrease in C3 level, thereby exacerbating the reduction in the generation of triglyceride. Three parts, including lower BMI, lower serum C3 and lower serum lipid level, might constitute a vicious spiral. Thus, C3 and ASP are increased in individuals with excess adiposity and with obesity [64]. Another study found that patients with BMI>35 kg/m2 took six weeks of very low calorie diet (VLCD), and it was observed circulating levels of C3 and BMI decreased [56]. Both of these confirm our speculation. In other words, we found the correlation between lipids and BMI. This could powerfully confirm that BMI is closely related to total body fat in our patients, and can also be used to reflect the nutritional status in our study. Underweight directly affects the circulating levels of C3 or temporarily inhibits C3 synthesis in the rest of the adipocytes, resulting in the increased incidence of hypoC3. Therefore, we hypothesize that underweight might affect renal outcome through its malnutrition status, and its tight association with C3.

Considering that lower weight might be the result of underlying diseases, we compared the frequencies of underlying conditions including hyperthyroidism and hepatitis B virus among different BMI groups. No association of these two diseases with BMI was found. Moreover, we found that inflammation markers including hsCRP and WBC increased when BMI increased. This finding suggests that baseline inflammation status is unlikely the reason for worse renal outcome in underweight IgAN patients. However, it remains possible that inflammation/infections during follow-up might be associated with the progression of IgAN. This hypothesis needs to be verified in further studies.

This study has several limitations. First, we only had 75 underweight patients in this study. The less recruitment of underweight patients would weaken the statistical power. Second, only baseline parameters including BMI were analyzed, and changes in these parameters may also be associated with renal function progression. Third, this is a single center study; and the results of this study still needs to be validated through further studies.

In this study, we found that underweight was associated with increased risk of renal function progression in IgAN by under-nutrition status and decreased C3 levels. It was also reasonable that additional protein wasting caused by chronic disease progression might be worse tolerated in the underweight group due to their low protein reserves. Furthermore, a positive correlation relationship between any two indicators from BMI, C3 and serum lipids was indicated. In the univariate Cox regression analysis, a decline in C3 is an independent risk factor for ESRD. Therefore, we hypothesize that underweight might reflect the decrease in lipid levels, which could directly lower C3 levels, in order to participate in the progression of IgAN in another aspect. These current findings emphasize the need for further confirming this risk factor. More mechanisms that contribute to underweight in patients with IgAN should be determined, and attempts should be made to ameliorate it.

## Supporting Information

S1 Table Distribution of underlying diseases in the four BMI groups. (DOC)
