# Supplementary material for: Underweight Is an Independent Risk Factor for Renal Function Deterioration in Patients with IgA Nephropathy
Source: PLoS One. 2016 Sep 9;11(9):e0162044. doi: 10.1371/journal.pone.0162044 (PMC5017745; doi:10.1371/journal.pone.0162044)
Supplement: S1 Table — (DOC) [file pone.0162044.s001.doc]

Supplement Table1. Distribution of underlying diseases in four BMI groups

| **Baseline variables** | **BMI (kg/m^2^)** | | | |
| --- | --- | --- | --- | --- |
|  | **<18.5** | **18.5-22.99** | **23-27.49** | **≥27.5** |
|  | **(N=75)** | **(N=394)** | **(N=345)** | **(N=116)** |
| Hyperthyroidism (N=3) | 0/75（0%） | 1/394（0.3%） | 1/345（0.3%） | 1/116（0.9%） |
| Hepatitis virus B carriers (N=37) | 4/75(5.3%) | 17/394(4.3%) | 14/345(4.1%) | 2/116(1.7%) |

Other three BMI groups compared with normal weight group, which was used as reference. *, P value <0.05; **, P value <0.01; ***, P value <0.001.
